# Swarm intelligence inspired shills and the evolution of cooperation

**DOI:** 10.1038/srep05210

**Published:** 2014-06-09

**Authors:** Haibin Duan, Changhao Sun

**Affiliations:** 1 State Key Laboratory of Virtual Reality Technology and Systems, Beihang University, Beijing 100191, P. R. China; 2 Science and Technology on Aircraft Control Laboratory, School of Automation Science and Electronic Engineering, Beihang University, Beijing 100191, P. R. China

## Abstract

Many hostile scenarios exist in real-life situations, where cooperation is disfavored and the collective behavior needs intervention for system efficiency improvement. Towards this end, the framework of soft control provides a powerful tool by introducing controllable agents called shills, who are allowed to follow well-designed updating rules for varying missions. Inspired by swarm intelligence emerging from flocks of birds, we explore here the dependence of the evolution of cooperation on soft control by an evolutionary iterated prisoner's dilemma (IPD) game staged on square lattices, where the shills adopt a particle swarm optimization (PSO) mechanism for strategy updating. We demonstrate that not only can cooperation be promoted by shills effectively seeking for potentially better strategies and spreading them to others, but also the frequency of cooperation could be arbitrarily controlled by choosing appropriate parameter settings. Moreover, we show that adding more shills does not contribute to further cooperation promotion, while assigning higher weights to the collective knowledge for strategy updating proves a efficient way to induce cooperative behavior. Our research provides insights into cooperation evolution in the presence of PSO-inspired shills and we hope it will be inspirational for future studies focusing on swarm intelligence based soft control.

Cooperation is omnipresent in real-world scenarios and plays a fundamental role for complex organization structures, ranging from biological systems to economic activities of human beings. However, it seems to be at variance with natural selection of Darwin's evolutionary theory^1^, which implies fierce competition for survival among selfish and unrelated individuals^2^. Hence, understanding the emergence and sustainability of wide spread cooperative behavior is one of the central issues in both biology and social science. This problem is often tackled within the framework of evolutionary game theory^3^,^4^,^5^. As the most stringent situation of reciprocal behavior through pairwise interactions, the prisoner's dilemma (PD) has long been considered as a paradigmatic example for studying the dilemmas between individual interests and collective welfare. In its original form, the PD is a two-player non-zero-sum game, where each player decides simultaneously whether to cooperate (C) or defect (D) without knowing *a priori* how its opponent will act. There are four possible outcomes for this game: (1) mutual cooperation (C, C) yields the largest collective payoff by offering each a reward *R*, (2) mutual defection (D, D) pays each a punishment *P*, and (3) the mixed choices (C, D) or (D, C) give the defector a temptation *T* and the cooperator the suck's payoff *S*^6^,^7^, with the payoff ranking satisfying *T*>*R*>*P*>*S* and 2*R*>*T* + *S*^8^. The dilemma is given by the fact that although the collective payoff would be maximized if both cooperated, it is best for a rational player to defect no matter what strategy its opponent chooses in a single round, making defection the only equilibrium. To allow the evolution of cooperation in the PD, suitable extensions have been proposed accordingly. One possible way out is direct reciprocity^9^ in the iterated prisoner's dilemma (IPD) game^10^, where players meet more than once, keep in mind the results of previous encounters and play repeatedly. Under this circumstance, reciprocity is regarded as a crucial property for winning strategies, including, but not limited to, *Tit for Tat* (TFT)^11^,^12^,^13^,^14^, *generous* TFT (GTFT)^15^, *win*-*stay lose*-*shift*^16^,^17^, to name but a few. Secondly, placing players on spatial networked structures, e.g., the square lattice, the small-world network, the scale-free network as well as diluted and interdependent networks^18^,^19^,^20^,^21^,^22^,^23^, has been acknowledged to be a new route to promotion and maintenance of cooperative behavior without any other assumptions or strategy complexity^24^. Though this is not universally true^25^,^26^, spatial interactions do provide cooperators an opportunity to agglomerate and grow, by which cooperators can finally resist exploitation by defectors and survive extinction in most cases^27^,^28^,^29^,^30^,^31^,^32^,^33^. Finally, other mechanisms favoring cooperation include co-evolution of the network structure along with playing rules^34^,^35^, the ability to move and avoid nasty encounters^36^, the freedom to withdraw from the game^37^,^38^, and punishment and reward^39^,^40^,^41^,^42^.

While the various mechanisms have achieved significant success in interpreting the evolution of cooperation, they are largely based on the assumption of particular networks of contacts and rules of local interaction. In other words, previous works mainly focus on which scenario favors cooperation. In the meanwhile, however, there exist many hostile scenarios in real life situations, where cooperation is disfavored and hence the undesirable outcomes need to be controlled for system efficiency improvement or disaster avoidance. For example, cooperative communications have been considered to be a promising transmit paradigm for future wireless networks, in which the interactions between neighboring nodes can be modeled via the evolution of cooperation. However, when the cost to cooperate exceeds a threshold, individual nodes tend to refuse to offer help to others, thus posing a great challenge to cooperation emergence as well as the performance of the whole system^43^. Generally, this problem becomes especially difficult when it is hard or even impossible to change the underlying playing rules of the original individuals, such as the behavior rules of crowds in a panic and the flying strategies of a flock of birds^44^. It is therefore of great interest and practical significance to design schemes that can effectively intervene in the collective behavior of a particular system, such that a proper equilibrium is guaranteed given the local rules of individual players and the network of contacts. Though many efforts have been devoted to pinning control law design and theoretical analysis of differential equations based control systems^45^,^46^, few literature have studied its application to complex systems of networked evolutionary games. To address this particular issue, Han *et al.* have proposed a new framework termed as soft control, which aims to induce desired collective behavior out of a multi-agent system via introducing a few controllable individuals, called shills, to the original population consisting of normal individuals^47^,^48^. Within the framework, shills are treated equally as normal agents by conforming to the basic playing rules, but are allowed to adopt elaborated strategies and updating rules for different mission objectives. Different from conventional distributed control that focuses on designing local rules for each agent in the network, soft control treats all the original individuals as one system and the control law is only for the shills. In real-world applications, there are two ways to account for the existence of shills: (i) under some certain circumstance, particular individuals of the original population turn into shills either voluntarily or forced by external factors, e.g., men of high principle who stand out in chaotic scenes to maintain order; (ii) additional individuals that are added to the original population to intervene in the macroscopic feature of the system, e.g., a few detectors who sneak into a band of gangsters with the purpose of making them confess^49^. So far, several preliminary proposals have been advanced to control the macroscopic behavior of multi-agent systems. For example, it has been proposed that a controllable powerful robot bird might be used as a shill to drive flocking of birds in airports, assuming the underlying dynamics of real birds follows Vicsek model^47^,^50^. Another work by Wang studied soft control in the well-mixed population case, where frequency-based tit for tat (F-TFT) was utilized as a shill's strategy^48^.

This paper goes beyond the updating rules of conventional evolutionary game theory and considers instead a swarm intelligence inspired strategy updating mechanism for shills. Generally, swarm intelligence refers to the collective behavior of a decentralized, self-organized natural system, such as a flock of birds trying to reach an unknown destination, a colony of ants searching for the best path to the food source, and a group of people brainstorming to come up with a solution to a specific problem^51^. Due to its decentralized distribution and self-adaption ability, swarm intelligence has provided a good source of intuition for solutions to realistic problems and several models have been proposed accordingly, among which are particle swarm optimization (PSO) proposed by Eberhart in collaboration with Kennedy^52^,^53^, ant colony optimization (ACO) by Dorigo^54^,^55^, and brain storm optimization (BSO) developed by Shi^56^,^57^,^58^. The fundamental concept of PSO is derived from the underlying rules that enable large numbers of birds to flock synchronously, where each individual in the swarm is seen as a particle, moving in a multi-dimensional space and searching for an unknown food source. Following random initialization of position and velocity vectors, each individual updates its state by combining one's past experience with some aspects of collective knowledge of the whole swarm in the following generations. As the searching process carries on, the population of particles, like a flock of birds foraging for food sources, move closer to and can eventually find the optimum of the utility function. Since miraculous swarm intelligence emerges from the simple and self-organized particles in PSO, we introduce the strategy updating mechanism to the shills and study whether a more desirable collective behavior could be induced in hostile environments. Furthermore, other than assuming a well-mixed population which may not be always true in real world systems, we extend soft control to a structured population, with each individual located on the vertex of a square lattice and its interaction restricted to the immediate neighbors along the social ties.

We address the above issue by introducing a model in which normal individuals interplay with a small fraction of shills and study what occurs depending on the frequency of each type of individuals and relative parameters tuning the updating procedure. To be specific, we choose a finite IPD game played on a square lattice as our strategic problem, where each individual adopts a stochastic strategy and plays according to Markov process during each evolution time step. All the details can be found in the Methods section. In what follows, we will focus on the influence of PSO inspired soft control on the evolution of cooperation under different settings by numerical simulations. Firstly, we start our study by varying the game strength from 1.1 to 2, in order to demonstrate that the proposed methodology takes effect upon cooperation promotion throughout a wide range of conflict intensity. Following this, we proceed to study the impact of strategy diversity resulting from soft control and find that PSO mechanisms enable evolutionary outcomes to be less dependent on random factors. Finally, we focus on the most unfavorable scenario of cooperation and study how the two crucial parameters, the shill fraction *p_s_* and the weighting coefficient *ω*, influence the evolution. We show that the frequency of cooperation can not only be promoted by shills, but can also be controlled to an expected value by choosing appropriate parameter settings.

## Results

Numerical simulations are carried out on an *L* × *L* square lattice with *N* = *L*^2^ vertices and equal connectivity <*z*> = 4. The results shown were obtained for communities of *N* = 10000 individuals. The equilibrium results, including frequencies of cooperation *f_c_* and average strategies <*x*>, are averaged over the last 10% time steps after a transient period of 300 time steps. This procedure is repeated 100 times for 100 independent random realizations of the game considered. The frequency of cooperation (or cooperation level) *f_c_* used to evaluate the system performance is defined as the percentage of Cs of all the actions taken by all individuals during one time step *t*. As there are 8*TN* actions in 4*N* *T*-IPD games, *f_c_* is calculated via 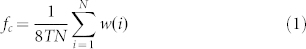
where *w*(*i*) denotes the number of Cs during the interactions between agent *i* and its four neighbors.

**Figure 1:**
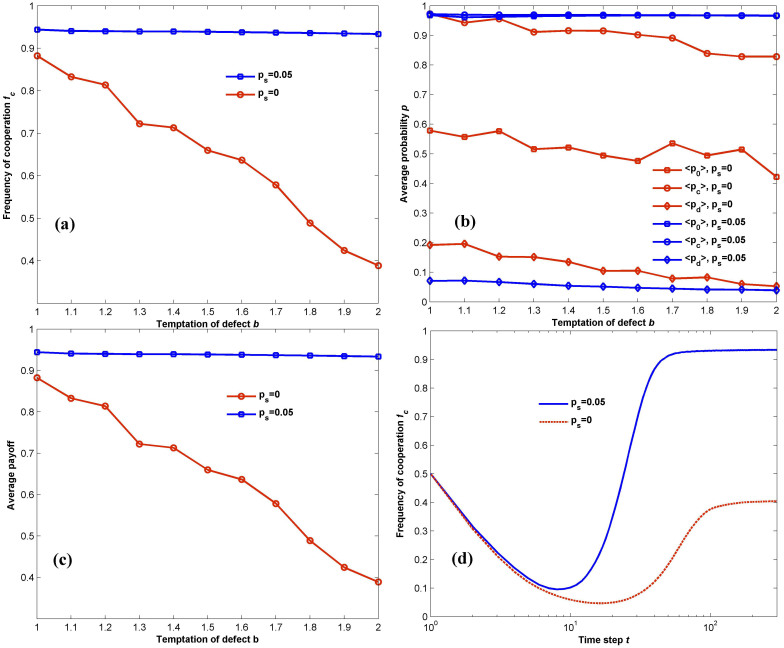
Comparison of evolution characteristics between cases with (*p_s_* = 0.05) and without shills (*p_s_* = 0). (a): average level of cooperation in dependence of *b*. (b): average Markov strategy of the population as a function of *b*. (c): average payoffs of one action in dependence of *b*. (d): evolution of cooperative behavior concentration over time step *t* for *b* = 2. We observe that while the spatial structure and iterated interactions contribute to the promotion of cooperation in PD games compared with one-shot games in a well-mixed population, soft control with a small proportion (*p_s_* = 0.05) of shills can further enhance cooperation levels even in strongly defection-prone environments, resulting in an average strategy of TFT. All trajectories are averaged over 100 independent realizations of the game considered, whose parameter setting is *L* = 100, *T* = 50, *ω* = 0.95, and the maximum evolution generation *G*_max_ = 300.

As shown in Figure 1(a), with a small fraction (*p_s_* = 0.05) of PSO-inspired shills introduced, the average cooperation level of the original population is greatly promoted, despite the fact that much fiercer conflict arises between individual interests and collective welfare when the temptation to defect *b* increases from 1 to 2. This should be largely attributed to the global search ability of PSO, by which the shills can find more successful strategies and spread them to the neighbors. In such a case, the temptation to defect *b* has a negligible effect on the value of *f_c_* in the equilibrium, which, as a matter of fact, only diminishes from 0.944 for *b* = 1 to 0.933 for *b* = 2. On the other hand, although spatial reciprocity and iterated interactions favor the emergence and maintenance of cooperation to some extent, they cannot guarantee a desirable level of cooperation in more defection-prone environments as the game intensity *b* grows. Expectedly and as shown by red circles in Figure 1(a), we observe a gradual decline of *f_c_*, which finally drops below the initial level (*f_c0_* = 0.5) of cooperation as *b* approaches its maximum limit of 2. It is also important to note that direct reciprocity emerging from iterated interactions does effect the evolutionary outcome, as we find that similar results cannot be obtained for one-shot IPD with *b* = 1.

As each individual adopts a stationary Markov strategy (*p*_0_, *p_c_*, *p_d_*) (see Methods), we examine the average state <*x*> = (<*p*_0_>, <*p_c_*>, <*p_d_*>) reached by the population in the equilibrium in Figure 1(b), each element of which denotes the average probability for a random individual to cooperate in the first stage of the IPD, the conditional probability to cooperate when the last move of the opponent is C or D respectively. For *p_s_* = 0.05 and 1<*b*<2, the individuals come to a consensus of Markov strategy approximate to (1,1,0), which can be viewed as TFT. Actually, TFT has been proven the simplest and the most successful strategy in the IPD game, via which one offers to cooperate initially, punishes defectors and reward cooperators in the successive rounds with “an eye for an eye”. Hence, desirable social welfare can always be achieved in such a case, as shown by blue squares in Figure 1(c). On the contrary, the original population without shills results in a worse strategy, in which one tends to cooperate with smaller probabilities as *b* grows. As a consequence, the average payoff an individual receives for each action diminishes from 0.9 to 0.4, as shown by the red circles in Figure 1(c). To have a better understanding of the evolution process, we plot the cooperation frequency as a function of time steps *t* in Figure 1(d) for both cases. Due to the random initialization for strategies, the evolutionary curves both start from *f_c_* = 0.5, an equal probability for each agent to cooperate or defect in the first generation. Similar to the cases in most literature^15^,^59^, the evolution courses follow the pattern of endurance and expansion: defectors take advantage of the random arrangements at the early stage and can thus make the greatest profits by exploiting cooperators. It enables defectors to spread across the population such that only little clusters of cooperators exist and a decline of *f_c_*is observed in the critical time step. Restarting from the lowest point, where an ordered distribution of cooperators and defectors is reached, clusters of cooperators begin to expand until a new equilibrium between cooperation and defection is achieved, so we see a rise of cooperation levels which gradually converge to a stable state.

**Figure 2:**
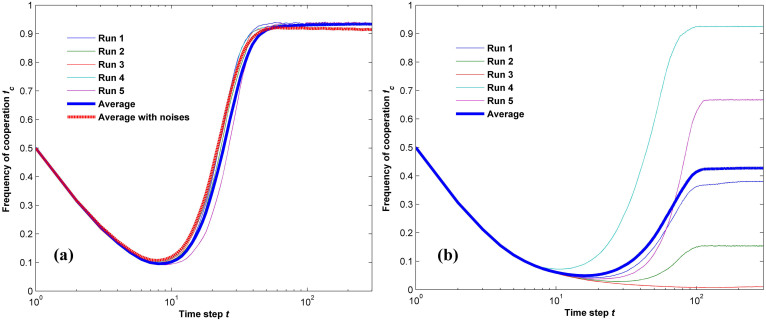
Evolution of the cooperation level over time steps with and without soft control in 5 random runs for b = 2. (a): *p_s_* = 0.05, *ω* = 0.95. (b) *p_s_* = 0. The blue, thicker trajectories indicate average performance of five simulation runs and the rest represent outcomes of 5 independent runs. The red dotted line in panel (a) shows the average cooperation frequency with noises included in the strategy updating process for shills. It can be observed that without soft control, the outcomes of one-shot run is dependent on randomness with a large value of the standard deviation. On the contrary, soft control not only makes the outcome undisturbed by randomness, but is also robust to noises in the updating process.

It is also found that, without soft control, the outcome of one random run of the simulation is more dependent on the initial strategy profile and its distribution, as is shown in Figure 2. For *p_s_* = 0, the stationary outcomes of five runs diverge significantly from the average performance value in Figure 2(b), whereas the dramatic deviation is removed by the proposed soft control mechanism with *p_s_* = 0.05 in Figure 2(a). This finding is easy to understand if we analyze the evolutionary dynamics of the two cases. On the one hand, as the players in the original population adopt the updating rule of *unconditional imitation* (see the Methods section for the detailed definition), which means strategies are produced by copying the old ones and thus no new acting tactics are created, the strategy diversity without soft control is highly dependent on the initial strategy profile. As a consequence, the outcome of one random simulation makes little sense in representing the evolution dynamics, for it is more sensitive to randomness. Yet, as more runs of simulations are carried out, the result gradually converges to a value which can be used to approximate the average cooperation level within a specific parameter setting. On the other hand, with PSO inspired soft control, new strategies are adaptively generated to maximize the potential payoff by considering simultaneously the most profitable strategy in the past and the best strategy of the neighbors, enabling the strategy variety to be less dependent of randomness brought about by the initialization operation. In sum, while promoting cooperation in strongly hostile environments, the proposed approach can lower the impact of random factors by adding to the strategy diversity in the population.

**Figure 3:**
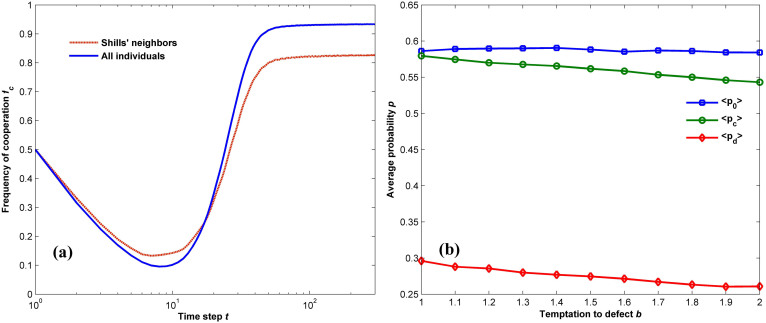
Evolution characteristics of shills. (a): evolution of the frequency of cooperation for different individuals for *p_s_* = 0.05, *ω* = 0.95, and *b* = 2. The blue line represents the frequency averaged over the whole population, while the red-dotted curve corresponds to the average cooperation of individuals within the neighborhood of shills. (b): average strategy of shills in the equilibrium as a function of the temptation to defect *b*. While shills promote the propagation of cooperative behavior across the network, relying more on history information (*ω* = 0.95) cannot maintain cooperation within their neighborhood.

The effect of shills in the boost of cooperation can be partly comprehended by the comparison between the evolution courses of cooperation frequencies for the whole population and the individuals within the neighborhood of shills. Focusing on Figure 3, we have two important findings: firstly, while both cases experience a decreasing phase of cooperation due to invasion of defectors in the early stage, the individuals adjoining to shills do maintain a higher level of cooperation; secondly, however, as cooperative behavior spreads with the evolution carrying on, the existence of shills hampers its further expansion. These results demonstrate that, although shills facilitate propagation of cooperation by exploring the strategy space, especially in the endurance period when the individuals are mostly defectors, the current parameter setting (*p_s_* = 0.05, *ω* = 0.95) cannot guarantee a global best strategy for shills in the equilibrium. Since the shills imitate the updating mechanism in PSO, the results can be interpreted as follows: unlike the normal agents who greedily switch their strategies to the best one within the neighborhood or remain unchanged, shills conduct effective search for potentially better strategies in a continuous 3-dimension space, which helps to increase the fraction of cooperation in shills and their neighbors in the early stage. On the other hand, as *ω* = 0.95 is used to balance the weight between one's best strategy in the history as well as the most profitable strategy within its neighborhood, shills tend to take into account more history information when updating strategies, which plays a less important role for payoff improvement in the current situation. As a result, while the average strategy of the whole population converges approximately to TFT, the shills result in an reacting rule <*x*> = (0.58,0.55,0.26) as shown in Figure 3(b), which makes the average payoff of individuals within shills' neighborhood lower than the average level of the whole system. Hence, we argue that, while shills facilitate the propagation of cooperative behavior across the network, relying more on history information (*ω* = 0.95) does not contribute to maintenance of cooperation within their neighborhood.

**Figure 4:**
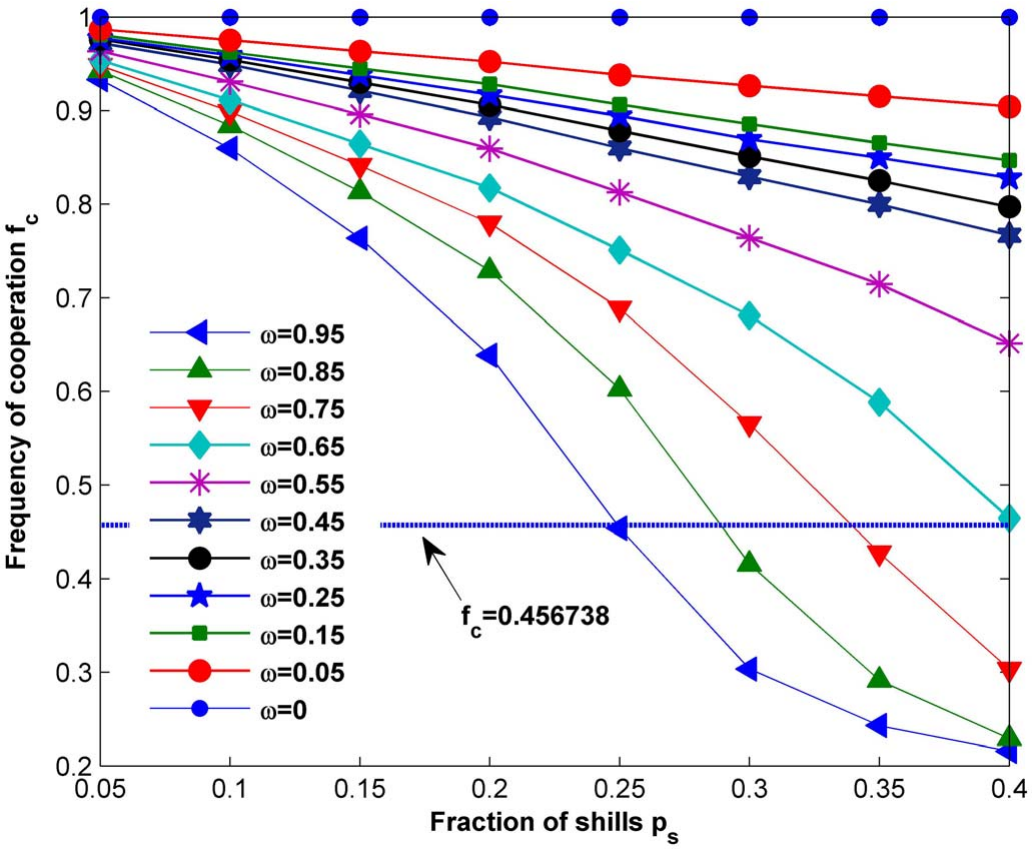
Dependences of cooperation frequencies on *p_s_* for different values of *ω* with *b* = 2. Each curve with markers shows the frequency of cooperation in the equilibrium as a function of *p_s_* for different *ω*, and the blue dotted line represents the average cooperation level *f_c_* = 0.456738, which is achieved by the original population without shills introduced. It can be observed that: (i) for all 0<*ω*<1, cooperation cannot be further promoted by adding more shills; (ii) for all *p_s_*<0.4, smaller *ω* facilitates the promotion of acooperation compared with larger values. We draw the conclusion that assigning higher weights to collective knowledge of the neighborhood swarm is a better choice in strategy updating for cooperation enhancement, whereas simply sticking to one's history memory results in low cooperation level. The results are averaged over100 independent realizations and the parameter setting is: *L* = 100, *T* = 50, *G*_max_ = 300.

**Figure 5:**
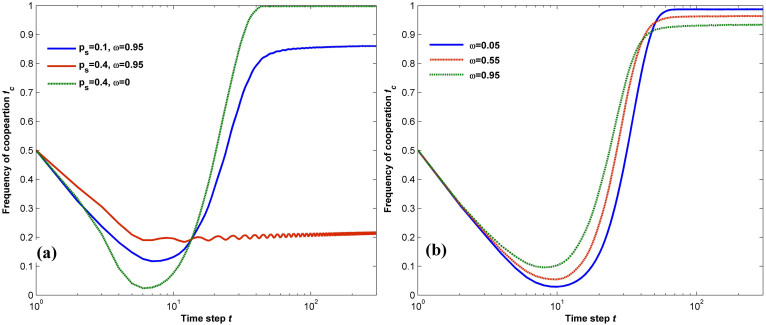
Evolution of cooperation for different *p_s_* and *ω* with *b* = 2. (a): frequency of cooperation as a function of time steps *t* with *ω* = 0.95. (b): frequency of cooperation as a function of *t* for *p_s_* = 0.05. Results show that the parameter setting of large *p_s_* and *ω* generates oscillation in the evolution course.

This reasoning is fully supported by the results shown in Figure 4, where we plot the dependence of the frequency of cooperation on both *ω* and *p_s_* with *b* = 2. For *p_s_*<0.25, all 0<*ω*<1 induces a higher level of cooperation compared to the average value *f_c_* = 0.4556738 achieved by the original population, while the promotion of cooperation is guaranteed only by *ω* not exceeding a threshold *ω_c_* for *p_s_*>0.25. This should be attributed to the different impacts of *ω* and *p_s_* on the evolutionary outcome. Firstly, for a fixed value of *ω*, adding more shills does not contribute to further enhancement of cooperation and there exists a moderate boundary value that warrants the best promotion of cooperation. When *p_s_* is small (e.g., *p_s_* = 0.05), the cooperation level is enhanced jointly by the few shills and the majority of normal agents, among whom the shills act as pioneers by exploring potentially profitable strategies and the normal agents within the neighborhood of shills spread the successful ones by unconditional imitation. Although more shills being incorporated adds to the strategy diversity of the fundamental population, it however slows down the strategy propagation at the same time, for the proportion of normal agents is reduced. Therefore, it becomes more difficult for the population to reach a consensus without effective diffusion of strategies by enough normal agents. For this reason, as *p_s_* grows from 0.05 to 0.4, it takes the network a much longer time to stabilize and the stationary state shows up as an equi-amplitude oscillation process as shown by the red line in Figure 5(a). However, the amplitude of the oscillation decreases as more neighborhood information is used when *ω* = 0 is chosen. It is also worthwhile to note that our results verify the judgment made by the authors in^48^, as they believe there will be a critical value of shill numbers to achieve the best soft-control goal. Secondly, for a given fraction *p_s_* of shills, smaller values of *ω* lead to stronger boosts of cooperation in contrast with larger ones as shown both in Figure 4 and Figure 5(b), suggesting that it benefits the population as a whole to learn from others in such a dynamical environment. In particular, *ω* = 0 can always results in the situation of global cooperation, in which *f_c_* = 1. This is different from the case in^6^, where all players follow the same updating rule of PSO and the most significant benefits are warranted by *ω* = 0.99 in scenarios strongly unfavorable for cooperation. The reason can be found when we compare our model of soft control with the optimization process using PSO. When searching for an optimal solution to a particular problem in the feasible space, each particle is faced with a static environment in term of the fitness function, i.e., each strategy corresponds to a fixed payoff. However, in the evolutionary game scenario, the payoff each individual receives depends on both its own strategy as well as the strategy of its opponent. In other words, the best strategy in the history is not necessarily a good choice for the current situation. In such a case, history information plays a less important role in updating strategies of shills towards the highest cooperation promotion of the population. On the other hand, the strategies in the neighborhood provide more useful information. As a consequence, assigning higher weights to the collective knowledge of the neighbors via small *ω* proves an effective way of inducing cooperation in such defection-prone environment.

## Discussion

In this paper, we have investigated the impact of PSO inspired soft control on the evolution of cooperation in a networked IPD game. Following the concept of soft control, we introduce shills into the original population without violating the underlying rules and guide the strategy updating of shills by PSO mechanisms, which make use of history information and the collective wisdom gained by the swarm to search for the most profitable strategy. Through intensive simulations, we demonstrate that the cooperation level can be controlled to particular values by selecting control parameters *p_s_* and *ω* appropriately. Specifically, we have shown that, on the one hand, it does not contribute to further cooperation promotion to add more shills but suppresses the propagation of cooperative behavior instead. Hence, we draw the conclusion that there exists an optimal boundary value guaranteeing the best promotion of cooperation for such an evolutionary network. On the other hand, we have found that relying more on the collective knowledge of the neighbors during strategy updating of shills always results in a stronger promotion of cooperation, while blindly sticking to one's history information hinders the emergence and sustainability of cooperation in the population. Besides, it is shown that the incorporation of shills enables the evolutionary outcome to be less dependent of random factors of the evolution.

As the first step to introduce swarm intelligence to soft control in a spatial evolutionary game, our research sheds some light on the role of PSO inspired shills in evolution dynamics of cooperation and provides a useful tool to intervene in collective behavior of self organized individuals, with the purpose of promoting cooperative behavior to a desirable level. Furthermore, this study can also be used to interpret the emergence of cooperation in a structured population of unrelated individuals, since special individuals acting like shills in soft control have been widely observed in natural systems. However, much work related to soft control remains to be carried out and our future work will focus on the following three aspects. In the first place, it is interesting to introduce other swarm intelligence mechanisms to soft control and study their impacts on cooperation evolution, such as ACO and BSO. Additionally, we will extend the networked structure to more realistic models and particularly study the dependence of the evolution of cooperation on the distribution of shills. Finally, we will conduct research on algebraic formulation and global dynamics analysis of evolutionary game with soft control.

## Methods

For the calculations, we consider an evolutionary *T*-stage IPD game located on an *L* × *L* square lattice with periodic boundary conditions, where each player occupies a site of the graph and interacts with its four nearest neighbors. Following a large portion of literature, we parameterize the payoff matrix of the one-shot PD by 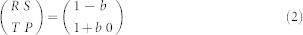
with *b* being the only free parameter to control the dilemma strength. Thus, the dilemma grows weaker as *b* approaches 0 and stronger confliction arises between individual interests and social welfare when *b* increases towards infinite. Note that *b* must be greater than 0 to conform to the definition of the PD game. Two types of individuals are involved in our model, namely normal individuals in the original population and shills added additionally for cooperation promotion, whose fractions are denoted by *p_n_* and *p_s_* respectively, satisfying *p_n_* + *p_s_* = 1. The initial distribution of shills is generated randomly and remains unchanged for the rest evolution duration.

Initially, each player is randomly assigned a stationary Markov decision strategy *x* = (*p*_0_, *p*_c_, *p*_d_)^8^,^60^, each element of which is drawn uniformly from the region between 0 and 1. Specifically, *p*_0_ is the probability for an agent to offer cooperation at the first stage, *p*_c_ and *p*_d_ denote respectively the conditional probability of an agent to cooperate when the its opponent cooperates and defects in the previous encounter respectively. It is worth mentioning that for each individual, the strategy does not change with time during each time step. The evolution is carried out by implementing a synchronous update process: at each time step *t*, every individual *i* accumulates its payoff 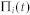 by means of pairwise interaction with all the neighbors in its neighborhood *U_i_*

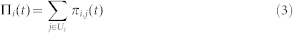
where *π_i,j_*(*t*) is the instaneous payoff of individual *i* playing a *T*-stage IPD game against its neighbor *j*; following this, each individual will simultaneously attempts to adapt its strategy with the purpose of maximizing its potential probability of success in future generations. Within the framework of soft control, shills are treated equally as normal individuals by complying to the basic playing rules for consideration of avoiding deception and exploitation by normal agents^48^. Only in one certain aspect are shills different from normal agents, i.e., shills are allowed to adopt elaborated strategies and updating rules, which is of the paramount importance for desirable collective behavior induction. Based on this, we assume that normal individuals follow the updating process of *unconditional imitation*^27^, where the best strategy in a normal individual's neighborhood is selected as the strategy at the next time step *t* + 1 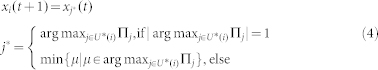
where set U*(*i*) includes individual *i* besides its neighbors and |**A**| denotes the element number of set **A**. On the contrary, shills follow a more complex updating mechanism, which is inspired by swarm intelligence emerging from bird flocks searching for food sources: each shill is considered to be a particle moving in a multi-dimensional space **R**[0,1]^3^, interacting with its neighbors, and adopting its strategy by combining some aspects of its memory as well as heuristic information from its neighbors. Specifically, each shill updates its strategy *x_i_*(*t*) following 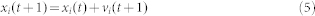

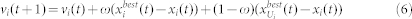
where *v_i_*(*t* + 1) is the velocity vector of agent *i* at time step *t* + 1, 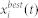 is the best-ever strategy of agent *i* throughout its history, and 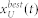 denotes the best strategy in its neighborhood at time step *t*. In our model, the initial velocity vector of each shill is randomly generated in the continuous 3-dimensional space **R**[−1,1]^3^. The weighting coefficient *ω* is utilized to keep a balance between one's own past information and swarm wisdom of the neighborhood. According to equations (5) and (6), a shill tends to stick to its own memory and make decisions based on its best ever strategy when *ω* is close to 1. Nonetheless, it takes into more consideration the swarm intelligence of its neighbors as *ω* approaches 0. In particular, a shill copies its best action in the past when *ω* = 1 and imitates the current best strategy in the neighborhood with the highest performance Π when *ω* = 0.

In the Results section, the proportion of shills *p_s_*, the game strength *b*, and the weighting parameter *ω* are considered as crucial parameters in investigating the evolution of cooperation under the framework of soft control.

## Author Contributions

All authors have made contributions to this paper. H.D. and C.S. designed the whole work, produced all the data, and wrote the paper. H.D. supervised the whole work and contributed to the manuscript preparation. C.S. made simulations. All authors discussed the results and reviewed the manuscript.
